# PVAmpliconFinder: a workflow for the identification of human papillomaviruses from high-throughput amplicon sequencing

**DOI:** 10.1186/s12859-020-03573-8

**Published:** 2020-06-08

**Authors:** Alexis Robitaille, Rosario N. Brancaccio, Sankhadeep Dutta, Dana E. Rollison, Marcis Leja, Nicole Fischer, Adam Grundhoff, Tarik Gheit, Massimo Tommasino, Magali Olivier

**Affiliations:** 1 International Agency for Research on Cancer, Lyon, France; 2 Department of Cancer Epidemiology, Moffitt Cancer Center, Tampa, Florida USA; 3 Institute of Clinical and Preventive Medicine, University of Latvia, Riga, Latvia; 4 German Center for Infection Research, Hamburg-Borstel-Lübeck-Riems, Hamburg, Germany; 5 Institute for Medical Microbiology, Virology and Hygiene, University Medical Center Hamburg-Eppendorf, Hamburg, Germany; 6 Heinrich Pette Institut, Leibniz Institut for Experimental Virology, Hamburg, Germany; 7 Department of Oncogene Regulation, Chittaranjan National Cancer Institute, Kolkata, India

**Keywords:** Amplicon sequencing, Virus discovery, Papillomavirus, Workflow, Phylogeny

## Abstract

**Background:**

The detection of known human papillomaviruses (PVs) from targeted wet-lab approaches has traditionally used PCR-based methods coupled with Sanger sequencing. With the introduction of next-generation sequencing (NGS), these approaches can be revisited to integrate the sequencing power of NGS. Although computational tools have been developed for metagenomic approaches to search for known or novel viruses in NGS data, no appropriate tool is available for the classification and identification of novel viral sequences from data produced by amplicon-based methods.

**Results:**

We have developed **PVAmpliconFinder**, a data analysis workflow designed to rapidly identify and classify known and potentially new *Papillomaviridae* sequences from NGS amplicon sequencing with degenerate PV primers. Here, we describe the features of **PVAmpliconFinder** and its implementation using biological data obtained from amplicon sequencing of human skin swab specimens and oral rinses from healthy individuals.

**Conclusions:**

**PVAmpliconFinder** identified putative new HPV sequences, including one that was validated by wet-lab experiments. **PVAmpliconFinder** can be easily modified and applied to other viral families. **PVAmpliconFinder** addresses a gap by providing a solution for the analysis of NGS amplicon sequencing, increasingly used in clinical research. The **PVAmpliconFinder** workflow, along with its source code, is freely available on the GitHub platform: https://github.com/IARCbioinfo/PVAmpliconFinder.

## Background

Papillomaviruses (PVs) are widely distributed across vertebrates. PVs are classified into genera, species, and types based on the nucleotide sequence identity of the major capsid protein L1. Human PVs (HPVs) have a tropism for the skin and mucosal epithelia of different anatomical sites and are organized into five major genera: alpha, beta, gamma, mu, and nu [1, 2]. HPV infection is responsible for various diseases, including several types of cancer [3, 4]. To date, more than 200 HPVs have been fully characterized [1, 5]. Recent studies have provided evidence that many more HPV types exist [6, 7]. Thus, it is important to comprehensively describe the family of HPV types and evaluate their role in human diseases.

Traditionally, single-step or nested PCR amplification using consensus or degenerate primers has been used for the identification and characterization of novel HPVs [8–10]. This approach is time-consuming and laborious and has limitations in terms of sensitivity, especially in samples with low viral DNA load or in the case of co-infections with multiple HPV types. More recently, several PCR-based strategies using degenerate primers have been combined with the use of next-generation sequencing (NGS) to characterize PV virome composition or to search for new viruses [11–13]. We have recently developed a novel approach that enabled the description of 105 putative new PV types in skin and oral samples [7]. This approach required the development of a specific bioinformatics workflow, because no existing tools were adapted to our protocol design. Several bioinformatics tools have been developed to analyze NGS data for the detection of viruses, but most of them are designed to analyze the virome composition of known viruses in clinical settings, or to discover new viruses from DNA or RNA shotgun sequencing [14–21].

Here, we describe a new bioinformatics workflow, PVAmpliconFinder, specifically designed to rapidly identify and classify known and potentially novel viruses from the *Papillomaviridae* family from amplicon NGS using degenerate PV primers. PVAmpliconFinder is based on alignment similarity metrics, but also considers molecular evolution time for improved identification and taxonomic classification of novel PVs. The final output of the tool includes a list of fully characterized putative new *Papillomaviridae* sequences together with a graphical representation of the relative abundance and diversity of HPV sequence diversity in the tested samples.

## Methods

**Fig. 1:**
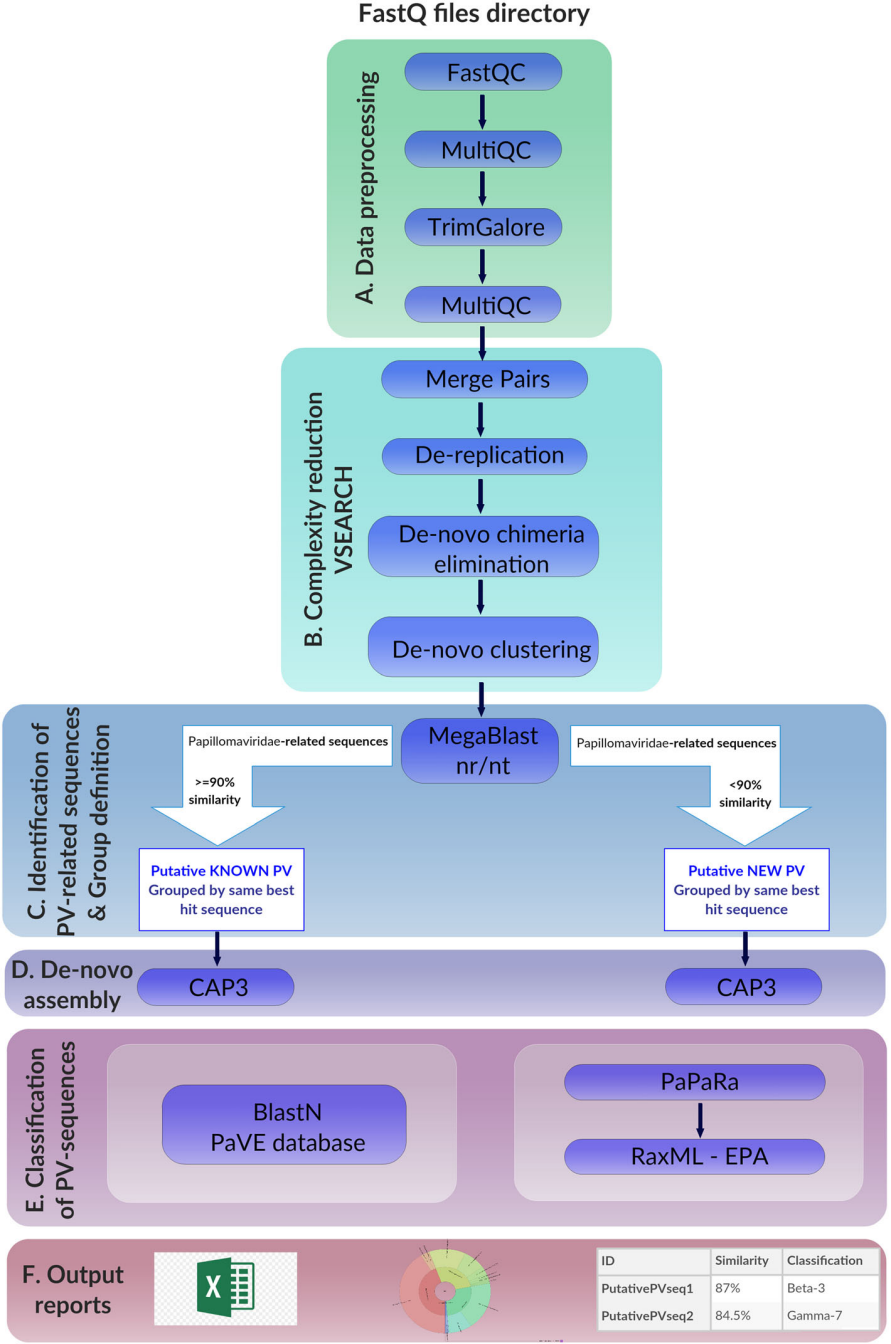
Workflow of PVAmpliconFinder

Details of the workflow can be found in Supplementary Data 2. Briefly, PVAmpliconFinder takes paired-end FastQ files as input and applies common data preprocessing steps for quality control and filtering (Fig. 1a). Then, data complexity is reduced before the identification of the PV-related sequences (Fig. 1b). Groups of sequences are defined based on similarity between identified sequences and available PV sequences in the NCBI database (Fig. 1c). De novo assembly is then performed to reconstruct the full amplified region covered by several primer systems (Fig. 1d). Finally, the reconstructed sequences are taxonomically classified based on two independent methodologies, which are alignment-based and homology-based, respectively, before the generation of diverse output reports (Fig. 1e and f).

## Results

We applied the PVAmpliconFinder workflow (Fig. 1) to the data obtained from amplicon sequencing of human skin swab specimens and oral rinses from healthy individuals, aiming to identify new PVs (the detailed protocol is in Supplementary Data 4). Different sets of degenerate primers targeting the L1 region of HPVs [7] were used to amplify 47 DNA samples and the amplification products were pooled in 8 DNA sample pools for sequencing (see detail in Supplementary Data 4). The 8 DNA sample pools were subjected to paired-end sequencing on the Illumina MiSeq system, generating about 2.65 million raw reads in total (331,359 raw reads on average per sample pool) (Table 1). PVAmpliconFinder was run with an info file describing the characteristics of each sample pool to enable the output of data stratified by tissue type and primer system (Supplementary Table S1).

**Table 1:** Summary of the number of individual sequences considered at each step of the workflow

Step	Total sequencing raw reads	TrimGalore		Merging		Dereplication		Chimeric identification		Clustering		Papillomaviridae best hit (eval < =1e-5)		Defined group (same best hit)			
														Putative new (> 10% dissimilarity)		Putative known (< 10% dissimilarity)	
Samples	N paired-end reads	N paired-end reads	%	N sequences	%	N sequences	%	N sequences	%	N sequences	%	N sequences	%	N sequences	%	N sequences	%
S1	564,435	564,064	99.93	551,266	97.73	22,551	4.09	22,498	99.76	79	0.35	61	77.22	0	0	5	8.20
S2	62,148	61,708	99.29	58,031	94.04	3281	5.65	3268	99.60	162	4.96	138	85.19	0	0	6	4.35
S3	316,297	315,999	99.91	307,400	97.28	15,562	5.06	15,562	100.00	51	0.33	49	96.08	1	2.04	18	36.73
S4	109,441	109,326	99.89	106,406	97.33	4842	4.55	4822	99.59	62	1.29	62	100.00	0	0	28	45.16
S5	309,779	309,390	99.87	294,563	95.21	14,101	4.79	14,091	99.93	140	0.99	129	92.14	2	1.55	39	30.23
S6	554,415	551,742	99.52	331,648	60.11	13,820	4.17	19,738	99.41	1162	8.46	910	78.31	0	0	16	1.76
S7	470,655	467,944	99.42	421,764	90.13	28,729	6.81	28,659	99.76	609	2.12	513	84.24	0	0	16	3.12
S8	263,707	263,270	99.83	244,177	92.75	13,293	5.44	13,283	99.92	194	1.46	188	96.91	0	0	8	4.26
Total number of sequences	2,650,877	2,643,443	99.72	2,315,255	87.58	116,179	5.02	115,921	99.78	2459	2.12	2050	83.37	3	0.45	136	16.73

### Preprocessing and complexity reduction analysis

The first step of the analysis, consisting of quality trimming, had a small impact on the total numbers of reads, removing less than 2% of the reads in the 8 DNA sample pools (Table 1). Merging the paired reads (step 2) reduced by at least two-fold the total number of sequences but extended their length. Although more than 90% of the reads were merged for most samples, about 40% of the reads were not successfully merged at this step for DNA sample pool 6 (Table 1). A quality check of this sample pool with the FastQC report generated in step 2 enabled the identification of primer contamination in about 10% of the reads, explaining a sub-optimal reconstruction of the full insert (data not shown).

The following step, de-replication, consisted of collapsing identical sequences into a single template but keeping the information on the number of reads used to form the final template. For the 8 DNA sample pools, the different amplicons were highly represented, as shown by the substantial decrease in the number of unique sequences remaining after this step (about 5% of the total number of sequences after merging of the mate reads) (Table 1). Less than 1% of the sequences were identified as potentially chimeric (Table 1). Then, a de novo clustering of highly related sequences was performed to correct for sequencing and/or polymerase errors present at low frequency at each position. A user-defined threshold had been set to 98% of identity for two sequences to cluster together. This clustering step drastically reduced the number of unique sequences retained, decreasing the number of sequences from about 8 to 1% of the overall sequences considered in the preceding step (Table 1). Overall, for the entire run, about 28.5% (756,506/2,650,877) of the total raw reads were retained for the MegaBlast step (Supplementary Table S5A).

### Identification of PV-related sequences and definition of groups

To identify the sequences in an unbiased manner, the sequences were aligned against the complete NCBI “nt” nucleotide sequence database, which includes all sequences from all species (Fig. 1c). Subsequently, groups of sequences were defined based on two characteristics: the best MegaBlast subject sequence for each query, and the percentage of similarity of each sequence with its corresponding best subject sequence (Fig. 1c).

#### Identification of PV-related sequences

On average, more than 90% of the centroid-clustered unique sequences of the 5 pools from skin swab specimens (S1-S5) matched against a *Papillomaviridae* family sequence, highlighting the specificity of the amplification using partially degenerate primers (Table 1). This represented a mean of 99.5% of *Papillomaviridae*-related reads among all reads submitted to MegaBlast from those 5 skin sample pools (Supplementary Table S5A).

For the 3 pools from oral rinses (S6-S8), about 86.5% of the centroid-clustered unique sequences had their best match against a *Papillomaviridae* family sequence (Table 1), representing a mean of 18.5% of *Papillomaviridae*-related reads among all reads submitted to MegaBlast from those 3 oral sample pools (Supplementary Table S5A).

A total of 549,280 reads (72.6%) of the sequences subjected to MegaBlast matched against *Papillomaviridae* family sequences (Supplementary Table S5A).

#### Definition of groups

When all the PV-related sequences identified above were grouped based on the best match and percentage of similarity, a total of 139 groups of PV sequences were found in the overall NGS run, including 136 known PVs or putative known PV variants (presenting less than 10% of dissimilarity with an already characterized PV) and 3 putative new PVs (Table 1). The known PV sequences corresponded to 549,273 raw reads, and the putative new PV sequences were supported by 7 raw reads (Supplementary Tables S5 E and G).

### De novo assembly of grouped sequences

The grouped sequences for each sample pool were then de novo assembled to extend the sequence lengths in order to cover the full L1 region targeted by the different primer systems used in the PCRs (Fig. 1d).

### Taxonomic classification of PV sequences

The taxonomic classification of each PV sequence was then assigned to the extended sequences using two methods, one based on the taxonomic classification of the best subject match (using the e-value computed by BlastN) when aligned against a comprehensive database of PV sequences, and the other based on molecular evolution using the Randomized Axelerated Maximum Likelihood-Evolutionary Placement Algorithm (RaxML-EPA) (Fig. 1e). For details, see Supplementary Data 2.

The results of the classification for DNA sample pool S5 (skin samples pool; CUT primer) are described in Table 2. In this sample pool, 2 putative new PV sequences represented by 5 reads and 39 putative known PV sequences represented by 60,892 reads were identified (Tables 1 and 2; Supplementary Tables S5 E and G).

**Table 2:** Taxonomic classification of Papillomaviridae-related reads from Sample 5

	Putative New			Putative Known		
N total sequences	N = 2 (5 reads)			N = 39 (60,892 reads)		
N sequence showen	N = 1 (3 reads)			N = 1 (4211 reads)		
	Megablast	BlastN	RaxML-EPA	Megablast	BlastN	RaxML-EPA
Alpha	–	–	–	–	–	–
Beta	–	–	–	–	–	–
Gamma	3 (100%)	3 (100%)	3 (100%)	–	4211 (100%)	–
Unclassified	–	–	–	4211 (100%)	–	4211 (100%)
Lambda	–	–	–	–	–	–
Tau	–	–	–	–	–	–

One of the putative new PV sequences in this pool was represented by 3 reads (PV_2). The MegaBlast algorithm (using the full “nt” database) aligned it against “Gammapapillomavirus 13 isolate Gamma13_HIVGc158, complete genome” (MF588722.1) with 81.25% of identity. Of note, although the Gamma13_HIVGc158 is a complete genome, this sequence is not reported in the Papillomavirus Episteme (PaVE) database. The BlastN algorithm (using the PaVE database) aligned this sequence against HPV-mEV03c45 (MF588721), an unreferenced Gamma PV genome, with 78.69% of identity. RaxML-EPA found the best position of this putative new sequence in the reference tree close to HPV213 (MF509818), also a potential Gamma PV, but with pending approval of its classification by the International Committee on Taxonomy of Viruses (ICTV) (Tables 2 and 3). Although the three methodologies agreed on classifying this sequence as a putative Gamma PV, the two alignment methods did not perfectly align the putative new PV sequence (less than 85% similarity against known PVs).

**Table 3:** Putative new *Papillomaviridae*-related sequences identified by PVAmpliconFinder

VIRUSname	PV_1	PV_2	PV_3	37VIRUSput
%dissimilarity	16.67	18.75	20.49	0.85
Abundance	0.0025	0.0049	0.0033	3.2918
N°reads	2	3	2	698
GInum	gi|1,273,499,301|gb|MF588716.1|	gi|1,273,499,348|gb|MF588722.1|	gi|270,048,224|gb|FJ969896.1|	gi|1,214,938,671|gb|MF356498.1|
AlignmentPosition_MegaBlast	3–78	1–352	207–327	1–236
VIRUS_closest_MegaBlast	Gammapapillomavirus 12 isolate Gamma12_EV07c367, complete genome	Gammapapillomavirus 13 isolate Gamma13_HIVGc158, complete genome	Human papillomavirus isolate GC04 major capsid protein L1 gene, partial cds	Human papillomavirus isolate ICB1, complete genome
Pool	pool3-skin-pathogen_S3_L001	pool5-skin-pathogen_S5_L001	pool5-skin-pathogen_S5_L001	pool4-skin-pathogen_S4_L001
Tissu	skin	skin	skin	skin
Primer	FAP	CUT	CUT	FAPM1
Length	160	353	372	262
AlignmentPositionBlastN_start:stop (length)	1–160 (160)	1–352 (352)	3–370 (368)	1–255 (255)
VIRUS_closest_Blast	HPV-mSK197(92.5%)	HPV-mEV03c45(78.69%)	HPV-mSK014(94.57%)	HPV224(97.25%)
BlastN_Classification	Gammapapillomavirus	Gammapapillomavirus	Gammapapillomavirus	Gammapapillomavirus
RaxML_closest_PV	HPV-mSK197	HPV213	HPV-mSK014	HPV224
RaxML_Classification	Gammapapillomavirus	Gammapapillomavirus	Gammapapillomavirus	Gammapapillomavirus
Sequence(s)	TAACAGTGGGCCACCCTTATTTCAGTGTTAAGAATGAAGGCACACAAGCCATAGTAGTTCCAAAGGTTTCAGGAGACCAGTTTAGAGTTTTCAGATTAAGACTCCCAGATCCTAACAAATTTGCTTTAATAGACCCATCTATATATAATCCAGAAAGAGA	GCCGGATCCGAATAAGTTTGCATTGATAGATCAGGACATTTATAATCCAGAAACGGAGAGATTAGTTTGGAGAGTTAAAGGCTTAGAGGTTGACAGAGGTGGTCCTCTAGGTATTGGAGCTGTAGGTCATCCTTTATTAAATAAATATGGAGATACAGAAAATCCTTTGGGAAGACCCATTCCAGAACAAGATGATAATAGAGTTAATTTATCGTTTGAACCAAAACAAACTCAAATTCTTATTGTTGGTTGTGCACCTCCTATAGGACAACATTGGGACGTTACAACACCTTGTAATAAACAGAATGCAGGCGAATGTCCACCTATAGCATTAAATCATACGAAAATACAGG	TGCCGGATCCGAATAAGTTTGCAATTGCAGATACTTGCTTGTATAATCCTGAAAAGGAGCGCTTGGTATGGCAGTTAGTGGGTTTAGAAGTTGACAGAGGTGGTCCTTTAGGAATTGGAGCCACCGGTCACCCATATTTCAATAAATATGTAGATACAGAAAATCCAGTAGCATATCCTCCAAAGCAAGAAGAAGCAGCATTAGATAGCAGGCAAGATATGTCCTTTGACCCTAAACAAGTACAAATGATAATTGTGGGCTGTGCACCTCCAACAGGAGAATATTGGGACACAACTAAATTTTGTGAATCTCATAAAAGTAGCCCAGGAGACTGTCCTGCAATAGAATTAATGCATACTATCATACAGGACG	TAACAGTTGGGCATCCTTATTTCAATAAAATTCAAGACACAGAAAACCCAAATAAATATGTGCCTAAAGCGGGCGATGAAAACAGATTAAATATTAGTGTTGATCCAAAACAGGTACAGCTACTTATTGTGGGCTGTGTGCCTGCAACAGGAGAACATTGGGATATTGCAAGGCCATGTGATGATGAGCAAAATGCTGGTGACTGTCCTCCTATCCAGCTTTTAAATACTGTAATTCAGGATGGCGATATGAGAGATATCGG

Among the 39 putative known PV sequences identified in this sample pool, one was represented by about 7% of the total reads (4211 raw reads out of 60,892 reads) (Table 2; Supplementary Table S6: Sequence identifier “69VIRUSput”). The MegaBlast algorithm aligned this sequence to a partial cds (342 bp) of a major capsid protein L1 gene (isolate GC12_1; FJ969907.1) with nearly 99% of identity. In comparison, the BlastN alignment against the PaVE database aligned this sequence against a Gamma-10 referenced PV genome (HPV130; GU117630), with a percentage of identity below 10% (86.12%). When aligning the isolate GC12_1 partial cds and the HPV130 full genome with the MegaBlast algorithm, the two sequences presented 86.01% of identity on 98% coverage. Finally, RaxML-EPA found homology with EdPV2 (MH376689), an unclassified *Erethizon dorsatum* PV species (Table 2; Supplementary Table S6). EdPV2 was proposed to represent a new genus in the family *Papillomaviridae* [22]. From these results, this 352 bp sequence may represent a novel PV type, although it remains to be fully characterized.

### Relative unnormalized abundance of *Papillomaviridae*-related sequence: differences based on the methodology

**Fig. 2:**
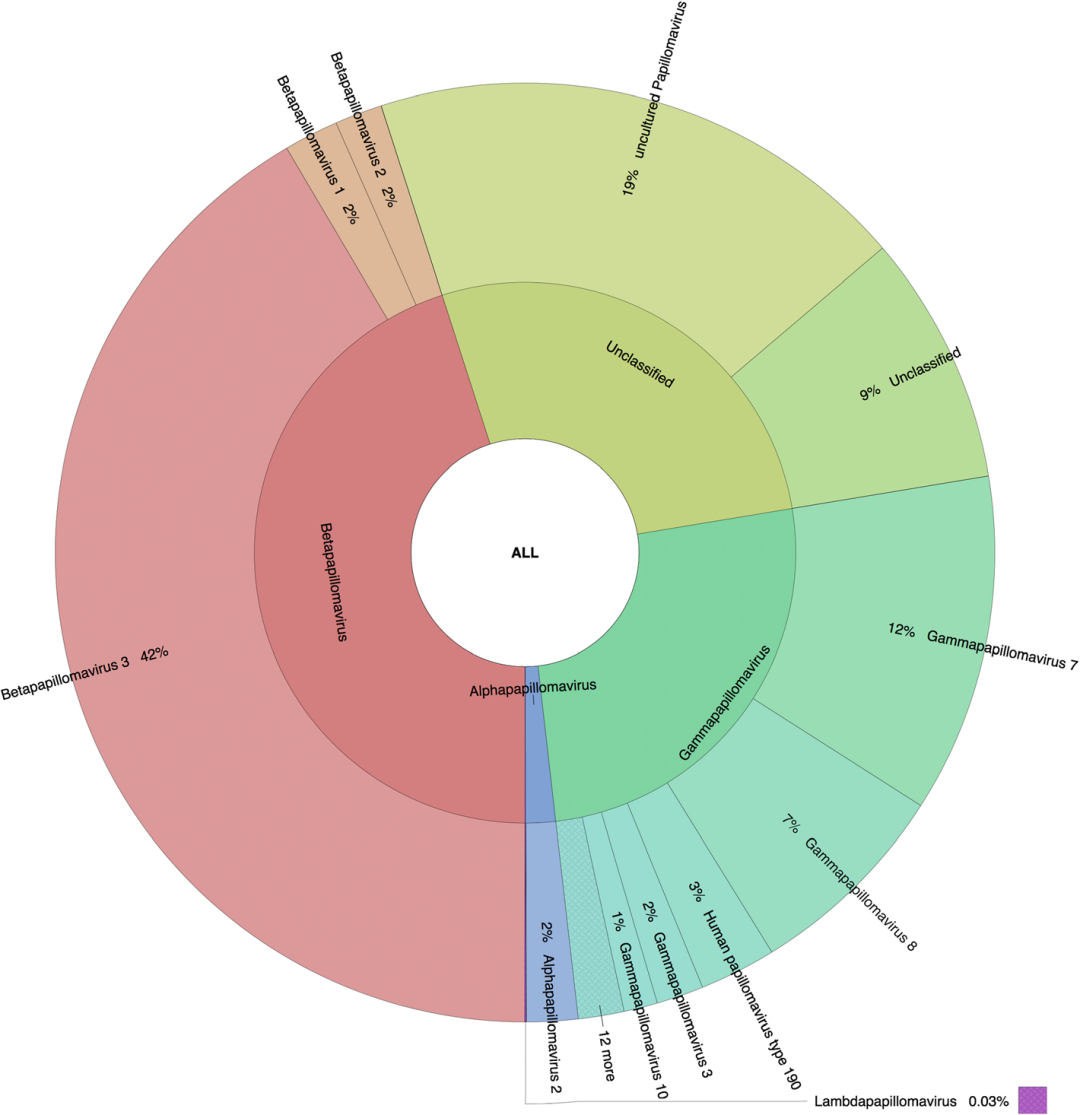
Graphical representation of the unnormalized abundance of PV genera and species in terms of number of reads based on MegaBlast alignment

**Fig. 3:**
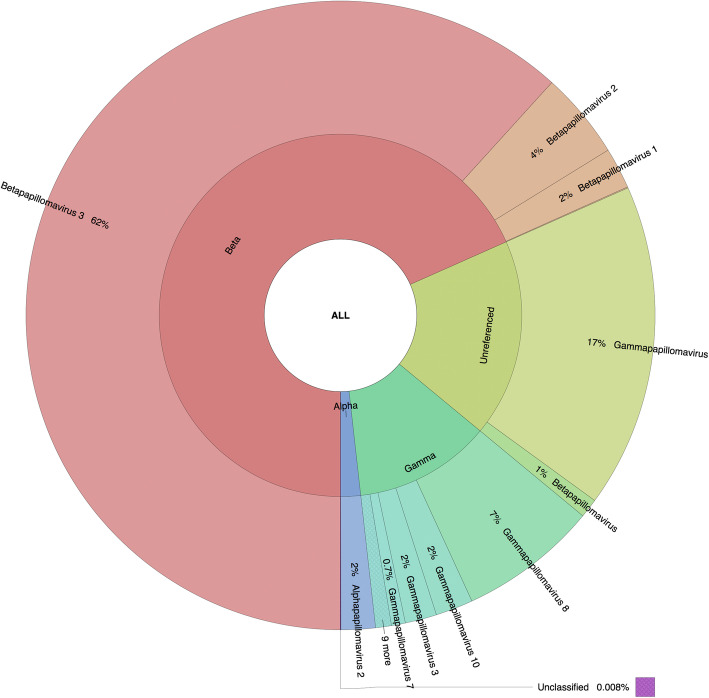
Graphical representation of the unnormalized abundance of PV genera and species in terms of number of reads based on BlastN alignment

**Fig. 4:**
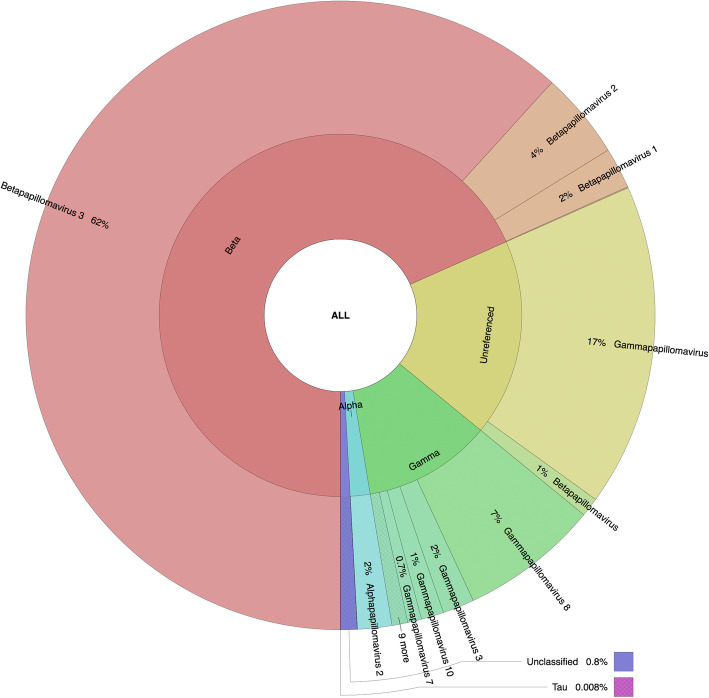
Graphical representation of the unnormalized abundance of PV genera and species in terms of number of reads based on RaxML-EPA

The relative unnormalized abundance of *Papillomaviridae*-related sequences identified by MegaBlast, BlastN, and RaxML-EPA for all samples is shown in Figs. 2, 3, and 4, respectively, and Supplementary Tables S2, S3, and S4 provide the detailed taxonomic assignation based on MegaBlast, BlastN, and RaxML-EPA, respectively. Beta-3 species were the most represented species identified by the three methods, with 42% of beta-3-related sequences identified by MegaBlast, and 62% identified by both BlastN and RaxML-EPA (Supplementary Tables S2, S3, and S4). The second most represented group was the “unclassified” sequences for MegaBlast (28% of the sequences), due to the incomplete taxonomic classification of a proportion of *Papillomaviridae-*related sequences present in the NCBI database. The third most represented genus based on MegaBlast was the gamma genus, with about 24% of the sequence, followed by the alpha genus (2%) and a small proportion of *Lambdapapillomavirus* (0.03%) due to the identification of a feline PV partial cds sequence (EF535004.1) in sample pools 1 and 2 (Supplementary Tables S2 and S6).

The second most represented group based on BlastN and RaxML-EPA was the unreferenced PVs, with a major subset putatively classified as unreferenced *Gammapapillomavirus* sequences (about 17%) and a small subset as unreferenced *Betapapillomavirus* sequences (about 1%). Of note, unreferenced sequences represented about 40% of the total entries available in the PaVE database version used (version of May 23, 2019). The third and fourth most represented genera were the referenced gamma and alpha PVs by both BlastN and RaxML-EPA (Figs. 3 and 4). BlastN could not classify 0.008% of the sequences, due to a best subject sequence associated with an e-value under the threshold defined as 1e-1 (Supplementary Table S3). RaxML-EPA also classified 0.8% of the sequences as “Unclassified” because those sequences presented homology to a newly described *Erethizon dorsatum* PV (EdPV2; MH376689), not yet classified by the ICTV, and potentially the first representative genome of a new PV genus [22]. Interestingly, the 46 reads that were unclassified by BlastN (due to the e-value threshold) were classified as *Taupapillomavirus* by RaxML-EPA, with homology to *Felis catus* PV type 4 and 5 (Supplementary Table S4).

### Discovery and characterization of putative new PV-related sequences

Overall, from the entire run, a total of 3 putative new sequences belonging to the *Papillomaviridae* family were identified by the algorithm (Table 3). Based on MegaBlast, “PV_1” is close to an unreferenced Gamma-12 complete genome, also present in the PaVE database (MF588716). However, it shows a higher percentage of identity with HPV-mSK197 (MH777339) based on BlastN alignment against the PaVE database. RaxML-EPA was in agreement with BlastN results, finding homology with the unreferenced *Gammapapillomavirus* HPV-mSK197. “PV_2” presented similarity (based on MegaBlast) with an unreferenced Gamma-13 complete genome (MF588722), which is absent from the PaVE database. BlastN found similarity with HPV-mEV03c45 (MF588721), an unreferenced *Gammapapillomavirus* genome, and RaxML-EPA found homology to HPV213 (MF509818), a referenced but unofficially classified *Gammapapillomavirus* genome. “PV_3” presented similarity with an unclassified partial cds of the isolate GC04 (FJ969896), but presented a higher similarity with the unreferenced HPV-mSK014 (MH777162) when aligned using BlastN. RaxML-EPA also found homology with the same HPV-mSK014 unreferenced *Gammapapillomavirus* genome. The sequence sizes ranged from 160 to 372 nucleotides, and all sequences presented more than 15% of dissimilarity with non-referenced PV sequences based on MegaBlast. All were amplified from skin DNA samples, using FAP and CUT primers [8, 9].

A previous analysis of the same data had led to the characterization of the full genome sequence of a novel Gamma-8 PV (Table 3, “37VIRUSput“) [23]. In the current analysis, this sequence appeared in the putative known PV sequence, because it is now included in the NCBI database (MF356498.1) as well as in the PaVE database. However, this sequence is still assigned to an unclassified group by the BlastN algorithm because the taxonomy has not yet been updated in the PaVE database (Supplementary Table S6, “37VIRUSput”). The official number of this novel PV, named “HPV isolate ICB1” in the NCBI database, is HPV224.

### Performances

The PVAmpliconFinder execution time on this dataset was less than 150 min when using an indexed NCBI database (Table 4). The most time-consuming step was the MegaBlast search against the full “nt” NCBI database (more than 95% of total time). When using a non-indexed NCBI “nt” database, the MegaBlast computational time was reduced to less than 5 min (Supplementary Table S7). For most of the steps, parallelization at the sample level was implemented to reduce the total computation time.

**Table 4:** Performance metrics of PVAmpliconFinder

Step	Total sequencing raw reads	TrimGalore	Merging	Dereplication	Chimeric identification	Clustering	MegaBlast	De-novo assembly	BlastN	RaxML	End
Time	+ 0′00”	+ 0′26”	+ 1′13”	+ 0′06”	+ 0′02”	+ 0′16”	+ 145′33”	+ 0′32”	+ 0′29”	+ 4′30”	+ 0′01”
Cumulative time	+ 0′00”	+ 0′26”	+ 1′39”	+ 1′45”	+ 1′47”	+ 2′03”	+ 147′36”	+ 148′08”	+ 148′40”	+ 149′10”	+ 149′11”

## Discussion

We developed PVAmpliconFinder, a complete workflow enabling the discovery and identification of viral sequences related to the *Papillomaviridae* family from targeted amplicon sequencing by NGS. PVAmpliconFinder is an easy single-line command workflow that takes FastQ files as input files and generates tabular and graphical output files that describe the nature and abundance of PV-related sequences present in a complex mixture of host, phage, bacterial, and viral DNA. The data output discriminates between putative new and previously known *Papillomaviridae*-related sequences. Furthermore, it includes sequencing metrics and sequence details, enabling the design of subsequent laboratory experiments for confirming the in silico findings (Supplementary Data 3).

In contrast to read-subtraction methods, PVAmpliconFinder performs an alignment step against the entire NCBI database. This is a deliberate choice because removing host sequences may remove potentially new viral sequences that present some similarity to the host. Indeed, viruses are the fastest mutating DNA element on Earth [24], so the chance of random sequence similarity between a large host genome and a small viral sequence is high. Moreover, the use of degenerate primer leads to the amplification of more diverse pieces of DNA and finding the best match against a *Papillomaviridae* sequence when aligning against a multi-organism database provides more robust results.

Several steps of the workflow are specifically tailored to deal with the specificity of NGS amplicon sequencing: the merging of the read pairs, enabling the reconstruction of the full insert; the de-replication step, to reduce data complexity and keep only one copy of identical sequences; and the elimination of chimeric sequences (PCR-derived sequences should be represented by at least two copies during the de-replication step; thus, single copies are probably sequences without biological significance). The number of de-replicated sequences corresponding to each template is saved in memory by the program to compute an unnormalized abundance. A step of clustering of highly related sequences is applied to correct for PCR amplification and sequencing errors. Because 2% of dissimilarity from any known L1 gene is enough to define a new PV variant [25], the tool uses a 98% identity threshold for clustering by default. When searching for new PV types (at least 10% of dissimilarity on the L1 gene), this threshold is a good compromise between sensitivity and specificity, because the potential loss of precision at the variant taxonomic level may be counterbalanced by an increased specificity of the reconstructed sequence.

To identify sequences in an unbiased manner, the sequences are aligned against the entire “nt” NCBI database. Although this step is time-consuming due to the large size of the database, it reduces the false-positive discovery rate. Indeed, querying a database with reduced diversity (such as a virus database) using the e-value as a threshold could increase the chances of getting a hit even if the subject sequence has a low identity with the queried sequence. Considering only the sequences that have their best match against a *Papillomaviridae* family sequence produces an unbiased result.

PVAmpliconFinder includes a grouping step to separate sequences that are putative new PVs from those that are already known PVs, using the threshold of 10% of dissimilarity. This grouping is done before the de novo assembly and classification steps because, although they are partially degenerate, the primers favor the amplification of known PV sequences. Because the tool is focused on the discovery of new PVs, it is important to separate potential new sequences at the earliest possible stage. A de novo assembly step is performed because of the possibility of using several primer sets that have different hybridization positions along the L1 gene. The objective is to reconstruct the longest possible sequence for each potential PV sequence.

PVAmpliconFinder uses an advanced identification and taxonomic classification of the sequences using both sequence similarity and homology. For the sequence similarity, the BlastN algorithm is used against the PaVE database [5]. This database is the most complete PV database. It includes PV sequences validated by full genome resequencing, but also several “non-referenced” genomes that are not classified taxonomically. Currently, non-referenced PV genomes in the PaVE database represent more than 37% of the overall available PV genomes (244/649), and this percentage continues to increase [26, 27]. PVAmpliconFinder presents the results based on the initial MegaBlast step and those obtained based on BlastN alignment against the PaVE database, but a huge number of sequences remain unclassified using the former approach because they match against incomplete L1 cds. Moreover, pairwise alignment with a low percentage of similarity raises a concern about the pertinence of the results obtained. This is especially true for the 3 putative new sequences identified in the application example reported here, because all sequences had at least 15% of dissimilarity against their best match. To circumvent this limitation, we use a complementary approach in parallel based on a molecular evolution method: RaxML-EPA [28]. A multiple sequence alignment is used to infer evolutionary time and to reconstruct a phylogenetic reference tree of selected species. Then, the Parsimony-based Phylogeny-Aware Read alignment (PaPaRa) algorithm is used to find the best position of the sequence into the reference multiple sequence alignment [29]. RaxML-EPA is subsequently used to find the best position of those sequences in the reference tree. The accuracy of the PaPaRa alignment is critical for the correct positioning of the query sequence into the reference tree.

Some limitations of the PVAmpliconFinder workflow are due to the inherent limitations of the methods implemented. Evolutionary based methods such as RaxML suffer from long-branch attraction errors. Long-branch attraction is an error where distant lineages are inferred to be close relatives because both have undergone a large number of changes. This is what is suspected to happen for the classification by EPA of the *Erethizon dorsatum* sequences identified in our experiment. They are inferred to be close to EdPV2 (MH376689), a recently referenced but unclassified *Erethizon dorsatum* PV [22], presenting large differences from other known PVs on its L1 gene, and thought to represent a new genus in the family *Papillomaviridae*. Although this led to an incomplete classification, these sequences may represent new species or virus features. Finally, PVAmpliconFinder does not control for potential contamination. Cross-contamination between samples during library preparation, amplification, and sequencing, or environmental contamination are difficult to detect using in silico methods. Low-abundance sequences may truly be present in the samples but may also come from cross-contamination from another sample. PVAmpliconFinder will report sequences represented by only 2 reads. These low-abundance sequences should be considered with caution. Defining an empirical abundance threshold could be considered. Environmental contamination may explain the presence of non-human PV in human samples. However, cross-contamination between species has recently been described [30, 31] and thus cannot be excluded.

While there is an increasing use of NGS amplicon sequencing in the clinical research setting, only few bioinformatics methods are available for the sensitive detection of HPV, and they are often restricted to a panel of already well characterized PV types [32]. The use of degenerated primers and PVAmpliconFinder may thus provide a solution for the detection and discovery of a broad range of HPV types.

In summary, we have developed the first bioinformatics tool for the identification of novel viruses of the *Papillomaviridae* family from amplicon sequencing data. This tool addresses a gap because no other tool exists for the analysis of this type of data. PVAmpliconFinder uses an advanced identification and taxonomic classification of the viral sequences extracted, which combines methodologies based on sequence similarity and homology. PVAmpliconFinder produces several tabular and graphical outputs that provide the necessary information to select the most promising putative new PV sequences that may be validated by further wet-lab approaches. Furthermore, PVAmpliconFinder can be easily modified and applied to other viral families, because this would only require a change in the interrogated databases and the reconstruction of a reference tree for the viral family considered. As no other tool exist for the analysis of NGS amplicon sequencing data of PV, PVAmpliconFinder addresses a gap with potential application in clinical research settings.

## Supplementary information

**Additional file 1: Supplementary Table S1.** Info file. **Supplementary Table S2**. Taxonomic classification of the reads identified in the overall NGS experiment by MegaBlast alignment. **Supplementary Table S3**. Taxonomic classification of the reads identified in the overall NGS experiment by BlastN alignment. **Supplementary Table S4**. Taxonomic classification of the reads identified in the overall NGS experiment by RaxML-EPA. **Supplementary Table S5**. NGS metrics, summary classification of putative known and putative new virus based on the three methodologies. **Supplementary Table S6**. Putative known *Papillomaviridae*-related sequences detected in the NGS experiment. **Supplementary Table S7**. Performances using non-indexed NCBI database

**Additional file 2: Supplementary Data 1.** Info file description. **Supplementary Data 2.** Details of the workflow steps. **Supplementary Data 3.** Description of output files format. **Supplementary Data 4.** Sample collection, preparation, and sequencing

## Data Availability

The PVAmpliconFinder workflow, along with its source code, is freely available on the GitHub platform: https://github.com/IARCbioinfo/PVAmpliconFinder. The datasets supporting the conclusions of this article are available in the NCBI database repository, under the BioProject accession number PRJNA555194.

## Abbreviations

cds: coding DNA sequence
EdPV2: *Erethizon dorsatum* Papillomavirus 2
HPV: Human PapillomaVirus
ICTV: International Committee on Taxonomy of Viruses
NCBI: National Center for Biotechnology Information
NGS: Next Generation Sequencing
PaPaRa: Parsimony-based Phylogeny-Aware Read Alignment program
PaVE: The PapillomaVirus Episteme
PCR: Polymerase Chain Reaction
PV: PapillomaVirus
RaxML-EPA: Randomized Axelerated Maximum Likelihood-Evolutionary Placement Algorithm
