# Supplementary material for: PVAmpliconFinder: a workflow for the identification of human papillomaviruses from high-throughput amplicon sequencing
Source: BMC Bioinformatics. 2020 Jun 8;21:233. doi: 10.1186/s12859-020-03573-8 (PMC7282039; doi:10.1186/s12859-020-03573-8)
Supplement: Supplementary file 2 — Additional file 2: Supplementary Data 1. Info file description. Supplementary Data 2. Details of the workflow steps. Supplementary Data 3. Description of output files format. Supplementary Data 4. Sample collection, preparation, and sequencing [file 12859_2020_3573_MOESM2_ESM.docx]

**Supplementary Data 1:** Info file description

This file should be a simple tabular text file (.txt or .csv) containing in the first column a character string corresponding to the FastQ file name upstream of the “R1” or “R2” tag (one line for one sample). The second column should be named “primer”, and should contain the information about the primer set used to amplify the L1 region (e.g. CUT or FAP) of the corresponding sample. The third and last column should be named “tissue” and should describe the sample source (e.g. skin or oral swabs). This information will be used during the creation of the output files and will help to distinguish the virome composition and the new target coming from different tissue types and amplified by different primer sets. If this option remains empty while the program is called, all the samples will be consider as coming from the same tissue type and being amplified with the same degenerate primers.

Supplementary Data 2: Details of the workflow steps

2.1 Input data type and format

The PVAmpliconFinder workflow is designed for the analysis of sequencing reads generated from paired-end sequencing of DNA amplified using degenerate primers targeting specifically the L1 sequence of papillomaviruses [1–3]. These primers enable the amplification of a region in the L1 gene out of a region of approximately 450 bp. The input data are FastQ files that can be uncompressed or compressed. The files will be automatically uncompressed if the detected format is a common compression format such as .zip, .gz, or .tar.gz. FastQ files from the forward and reverse reads of the same sample should have the same name, with only “R1” and “R2” differentiating the two files.

2.2 Input parameters

Three mandatory input parameters must be set: the path to the input directory that contains the FastQ files; a tag corresponding to the suffix of the FastQ file names to be selected for the analysis in the input directory; and the path to the output directory where the output files will be written. The following optional input arguments can also be set: [1] the name of the identifier of the NCBI “nt” database to be used (the Blast database should be present in the environment, and the default value is “nt”); [2] the number of threads to be used for the analysis (the default value is 2); [3] the directory path of an info file containing information on sample type and primer used (see Supplementary Table 1 for an example, and Supplementary Data 1 for how to format the file); [4] the threshold for the percentage of identity to be used in the *de novo* centroid-based clustering (the default value is 98).

2.3 Data preprocessing

The preprocessing of the FastQ files includes an initial quality control (QC) of the raw FastQ files using FastQC [4] and the aggregation of the FastQC reports using MultiQC [5] (Figure 1A). FastQ files are then trimmed for adapter sequences and sequences of amplification primers if required, using TrimGalore [6]. This step also discards low-quality bases, sequences of less than 32 bp, poly-A sequences, and reads with low average quality score. FastQC and MultiQC are run on the trimmed FastQ files for a final QC (Figure 1A).

2.4 Complexity reduction and removal of artifacts

The step’s aim is to eliminate the redundant sequences generated during the different PCR steps preceding sequencing and to correct sequencing and/or polymerase errors. Four modules from the existing tool VSEARCH [7] are used to perform three different steps, as described below (Figure 1B).

**2.4.1 Merging of reads**

The ”fastq_mergepairs” module merges Read 1 and Read 2 pairs and reconstructs the full amplicon (around 450 bp);

**2.4.2 De-replication of reads**

The “derep_fulllength” module de-replicates reads by keeping only one template of several identical sequences. This step is particularly important because duplicates are generated during the PCR amplification steps used to amplify the L1 region as well as for the pre-sequencing processing of samples.

**2.4.3 Chimera detection**

The “uchime_denovo” module is then run to identify and remove chimeric DNA sequences that often form during PCR amplification, especially when sequencing a unique region. The option –minuniquesize is used with 2 as default value to account for the fact that at this step each of the sequences is expected to be represented by at least 2 raw sequencing reads, corresponding to a minimum of one PCR cycle.

**2.4.4 Reduction of amplification artifacts**

The “cluster_size” module consists of *de novo,* centroid-based clustering of the sequences sharing more than a user-defined level of identity: 98% is the default value. This unique sequence will be used for downstream analysis. 2% of dissimilarity from any known L1 gene is enough to define a new PV variant [8]. When searching for new PV types (at least 10% of dissimilarity on the L1 gene), 98% of identity enables a good clustering to balance between sensitivity and specificity.

2.5 Identification of PV-related sequences

All sequences identified by the preceding metagenomic analysis are subject to a MegaBlast alignment against the full “nt” nucleotide collection from the NCBI database (default parameters) [9]. All sequences that have their best hit against any sequence belonging to the *Papillomaviridae* family with an e-value smaller than or equal to 1e-5 are kept for the next steps of the workflow (Figure 1C). *Papillomaviridae*-related sequences are identified using a lineages file created using the “ncbitax2lin” tool (10) (<https://github.com/zyxue/ncbitax2lin>).

2.6 Classification of PV sequences

This step uses two different approaches based on two different tools, BlastN and Randomized Axelerated Maximum Likelihood-Evolutionary Placement Algorithm (RaxML-EPA) [11], and the results of both approaches are returned. With RaxML-EPA, a method based on molecular evolution, a full taxonomic classification of the putative new sequences is obtained based on the homology of each sequence to its closest taxon. In both approaches, PV sequences are first grouped based on both the best MegaBlast subject sequence for each query and the percentage of similarity of this sequence with its corresponding best subject sequence. Then, a *de novo* assembly of sequences formed by this “two-features” grouping is performed with CAP3 [12] to reconstruct the full PCR amplicon because the different primers systems used are not targeting exactly the same L1 region. Finally, a taxonomic classification is performed on the reconstructed sequences (Figure 1C and Figure 1D), as detailed below.

2.6.1 Definition of groups and *de novo* assembly

For each sample, the sequences that have their best MegaBlast hit against a sequence belonging to the *Papillomaviridae* family are kept for the analysis. These sequences are grouped if their best hit is the same subject sequence. Subsequently, the grouped sequences are split into two groups: [1] putative known PVs, corresponding to sequences that present less than 10% of dissimilarity on their aligned portion with a known PV; [2] putative new PVs, corresponding to sequences that present more than 10% of dissimilarity on their aligned portion with a known PV. A *de novo* assembly is then performed for each group with CAP3 with default parameters (12) for contigs reconstruction (Figure 1C and Figure 1D).

2.6.2 BlastN-based taxonomical classification

Each contig sequence reconstructed during the previous step is then classified based on the taxonomic classification of its best alignment (BlastN best match) against the full L1 gene nucleotide sequence database available in the Papillomavirus Episteme (PaVE) database, the most comprehensive database of PVs [13] (Figure 1E). This step mimics the L1 taxonomic tool of the PaVE database (L1 Taxonomic tool, 1). The PaVE database provides full papillomavirus genome sequences with complete taxonomic classification (referenced PV), as well as full genomes with incomplete taxonomic classification (unreferenced PV). Referenced genomes correspond to genomes validated and fully characterized by the re-sequencing of the entire genome. Unreferenced genomes are mostly genomes identified through metagenomics approaches and submitted to PaVE but not validated for accuracy or novelty of the PV [14].

2.6.3 RaxML-EPA-based taxonomical classification

A reference phylogenetic tree (reference tree; RT) was constructed based on the full-L1 ORF nucleotide sequences of 597 available PV genomes retrieved from the PaVE database (https://pave.niaid.nih.gov/) in June 2019 [13]. The sequences were aligned at the nucleotide level using the MUSCLE algorithm, with the default parameters [15] in MEGA7 [16]. The final full-length L1-ORF alignment encompassing 597 full L1-ORF nucleotide sequences, 2913 positions, and 468 distinct alignment patterns constitutes the reference multiple sequence alignment (MSA). MEGA7 was used to test the best substitution model and for the phylogenetic inference. The codon positions included were 1st + 2nd + 3rd + non-coding. Based on the alignment using MUSCLE, all positions with <95% site coverage were eliminated (partial deletions), to enable the inclusion of taxa with some missing data. There were a total of 1383 positions in the final dataset.

A discrete gamma distribution was used to model evolutionary rate differences among sites (five categories; +G, parameter = 0.658). The rate variation model allowed for some sites to be evolutionarily invariable ([+I], 0.019% sites). The initial trees for the heuristic search were obtained automatically by applying the neighbor-joining (NJ)/BioNJ algorithm to a matrix of pairwise distances estimated using the maximum composite likelihood (MCL) approach and then by selecting the topology with the highest log likelihood value (-468961.607). The final tree selected constitutes the RT.

Phylogenetic inference was performed with MEGA7 using the general time-reversible (GTR) model of nucleotide substitution and 500 bootstrap replicates [17].

The Parsimony-based Phylogeny-Aware Read alignment (PaPaRa) program [18] algorithm is used to align each contig sequence, reconstructed during the previous *de novo* assembly step, against the MSA [19] (Figure 1E). Subsequently, the evolutionary placement algorithm (EPA) [20] in RaxML [11] is run to place the sequences into the RT (Figure 1E), based on PaPaRa multiple alignment. The EPA is run using the same nucleotide substitution model used to infer the reference phylogenetic tree. A script was developed in-house to parse the output format of the EPA [21] to extract, for each reconstructed sequence, its closest related taxon in the phylogenetic tree, and use this taxon to assign a taxonomic classification.

2.7 Output reports

Several output reports are generated as Excel files, fasta files, or graphical images from the different steps of the workflow. They describe summary sequencing statistics, the sequences of known or putative new PVs, the relative unnormalized abundance of PV types, and the taxonomic classification of all identified PV sequences. The use of an info file providing sample characteristics enables the output of statistics stratified by these characteristics (Figure 1F, Supplementary Table 1). The detailed list of files and file contents is available in Supplementary Data 3.

2.8 Performance testing

The performance of the bioinformatics workflow has been estimated on a computer with an Intel® Core™ i7-6700 processor CPU @ 3.40GHz × 8, 64 bits, 62.9 GB RAM, 256 GB SSD, in the Linux environment (Ubuntu 16.04 LTS).

Supplementary Data 3: Description of output files format

The output files generated by PVAmpliconFinder are:

- an Excel file named “Table_Summary_MegaBlast” (Supplementary Table 5) that contains several tables providing sequencing metrics by sample, primer set, or tissue type, and a classification of putative new and known PVs found in the samples, based on MegaBlast, RaxML, and BlastN results.

- several Excel file(s) containing a full taxonomic classification of the species present in the samples based on MegaBlast, BlastN, or RaxML-EPA results, with unnormalized relative abundance estimated as number of reads (Supplementary Tables 2, 3, and 4, respectively). Several tables are created if several tissue types have been specified in the info file, for the three methodologies applied to classify the PV sequences, and the information about primer used to detect the species is present (if also specified in the info file).

- a KRONA [22] graphical representation of the unnormalized abundance of PV genera and species, taxonomically classified based on MegaBlast, BlastN, and RaxML-EPA results, in terms of number of reads (Figure 2, 3 and 4, respectively). If an info file was provided as input, a graphical representation is produced for each tissue type, as well as an overall representation mixing the different tissue types.

- an Excel file named “Table_putative_known_PV”, containing the putative known PV sequences detected in the different samples (Supplementary Table 6). This file contain information such as: a unique identification for the sequence or the cluster of sequences corresponding to a putative known PV; the percentage of dissimilarity on the aligned portion of the sequence returned by MegaBlast (if several sequences in the cluster, the percentage of dissimilarity of the longest sequence is reported); the relative unnormalized abundance of the sequence(s) into the overall reads generated for the sample; the absolute number of reads used to generate the sequence(s); the GI number from MegaBlast rent; the closest PV species given by the BlastN against the PaVE database; the taxonomic classification at the genus level given by the BlastN against the PaVE database; the closest PV species given by the RaxML-EPA algorithm; the taxonomic classification at the genus level given by the RaxML-EPA algorithm; and the nucleotide sequence(s).

- an Excel file named “Table_putative_new_PV”, containing the putative new PV sequences detected in the different samples (Table 3). This file contains the same information as the “Table_putative_known_PV” Excel file described above.

- fasta files of the putative known and putative new PVs, named “Putative_known_PV.fa” and “Putative_new_PV.fa”, respectively. If a cluster contains several sequence, the sequences are attributed a unique incremental number after the unique name of the cluster (e.g. if there are 3 sequences in the cluster named “PV_1”: >PV_1.1, >PV_1.2, >PV_1.3).

Supplementary Data 4: Sample collection, preparation, and sequencing

Skin swab specimens (n = 25) were randomly-selected baseline samples from the VIRUSCAN Study, an ongoing five-year (2014–2019) prospective cohort study conducted at Moffitt Cancer Center and the University of South Florida (R01CA177586-01; “Prospective study of cutaneous viral infections and non-melanoma skin cancer”).

In addition, oral rinses (n = 22) were randomly selected from a pilot study that aimed to estimate the prevalence of Helicobacter pylori in oral gargles from a Latvian population. The study was approved (No. 8-A/15) by the Ethics Committee of Riga East University Hospital Support Foundation.

After DNA extraction, all samples were processed at the International Agency for Research on Cancer (Lyon, France). PCR protocols were used as previously described in [23]. Briefly, we used the original FAP and CUT primers that target the L1 gene [1,3], two improved broad-spectrum FAP primer systems that show an increased specificity for beta HPV types, as well as two primers systems aligning to the L1 open reading frame (ORF) of all four beta-3 HPV types (HPV49, 75, 76, and 115). Each of the six primer systems used (FAP, FAPM1, FAPM2, CUT, Beta3-1, Beta3-2) enables the amplification of a region in the L1 gene of approximately 450 bp (all the primers sequences have been provided in [23]).

In total 47 DNA samples were amplified according to different PCR protocols explained above. PCR amplicons were visualized by electrophoresis on a 2% agarose gel and purified using QIAquick gel extraction purification kit according to the manufacturer’s instructions (QIAGEN, Hilden, Germany). Purified PCR amplicons were divided into height different pools from which were prepared libraries using the NEBNext Ultra DNA library prep kit and MiSeq reagent kit version 2 (Illumina). Each NGS pools included purified amplicons generated using the same PCR protocol and distributed as followed : 5 skin DNA samples in DNA sample pool 1 (Beta3-1), 5 skin DNA samples in DNA sample pool 2 (Beta3-2), 5 skin DNA samples in DNA sample pool 3 (FAP), 5 skin DNA samples in DNA sample pool 4 (FAPM1), 5 skin DNA samples in DNA sample pool 5 (CUT), 5 oral DNA samples in DNA sample pool 6 (FAPM1), 12 oral DNA samples in DNA sample pool 7 (FAPM2), 5 oral DNA samples in DNA sample pool 8 (CUT). Paired-end NGS sequencing was performed using an Illumina MiSeq (600 cycles). The final mean read size was 227 bp. Demultiplexing was done using Illumina internal software CASAVA v1.8.2. The SRA accession number of the data is PRJNA555194.

**References**

1. Chouhy D, Gorosito M, Sánchez A, Serra EC, Bergero A, Fernandez Bussy R, et al. New generic primer system targeting mucosal/genital and cutaneous human papillomaviruses leads to the characterization of HPV 115, a novel Beta-papillomavirus species 3. Virology. 2010;397:205–16.

2. Forslund O, Antonsson A, Nordin P, Stenquist B, Göran Hansson B. A broad range of human papillomavirus types detected with a general PCR method suitable for analysis of cutaneous tumours and normal skin. Journal of General Virology. 1999;80:2437–43.

3. Forslund O, Ly H, Higgins G. Improved detection of cutaneous human papillomavirus DNA by single tube nested ‘hanging droplet’ PCR. Journal of Virological Methods. 2003;110:129–36.

4. Andrews S. FastQC: a quality control tool for high throughput sequence data. 2010. https://www.bioinformatics.babraham.ac.uk/projects/fastqc/.

5. Ewels P, Magnusson M, Lundin S, Käller M. MultiQC: summarize analysis results for multiple tools and samples in a single report. Bioinformatics. 2016;32:3047–8.

6. Krueger F. Trim Galore! : A wrapper tool around Cutadapt and FastQC to consistently apply quality and adapter trimming to FastQ files, with some extra functionality for MspI-digested RRBS-type (Reduced Representation Bisufite-Seq) libraries. 2015. https://www.bioinformatics.babraham.ac.uk/projects/trim_galore/.

7. Rognes T, Flouri T, Nichols B, Quince C, Mahé F. VSEARCH: a versatile open source tool for metagenomics. PeerJ. 2016;4:e2584.

8. Bernard H-U, Burk RD, Chen Z, van Doorslaer K, Hausen H zur, de Villiers E-M. Classification of papillomaviruses (PVs) based on 189 PV types and proposal of taxonomic amendments. Virology. 2010;401:70–9.

9. Altschul SF, Gish W, Miller W, Myers EW, Lipman DJ. Basic local alignment search tool. J Mol Biol. 1990;215:403–10.

10. Mahmoudabadi G, Phillips R. A comprehensive and quantitative exploration of thousands of viral genomes. eLife. 2018;7:e31955.

11. Stamatakis A. RAxML version 8: a tool for phylogenetic analysis and post-analysis of large phylogenies. Bioinformatics. 2014;30:1312–3.

12. Huang X, Madan A. CAP3: A DNA Sequence Assembly Program. Genome Res. 1999;9:868–77.

13. Van Doorslaer K, Tan Q, Xirasagar S, Bandaru S, Gopalan V, Mohamoud Y, et al. The Papillomavirus Episteme: a central resource for papillomavirus sequence data and analysis. Nucleic Acids Res. 2013;41:D571–8.

14. Simmonds P, Adams MJ, Benkő M, Breitbart M, Brister JR, Carstens EB, et al. Consensus statement: Virus taxonomy in the age of metagenomics. Nature Reviews Microbiology. 2017;15:161–8.

15. Edgar RC. MUSCLE: multiple sequence alignment with high accuracy and high throughput. Nucleic Acids Res. 2004;32:1792–7.

16. Kumar S, Stecher G, Tamura K. MEGA7: Molecular Evolutionary Genetics Analysis Version 7.0 for Bigger Datasets. Mol Biol Evol. 2016;33:1870–4.

17. Nei M, Kumar S. Molecular Evolution and Phylogenetics. Oxford University Press; 2000.

18. Berger SA, Stamatakis A. PaPaRa 2.0: A Vectorized Algorithm for Probabilistic Phylogeny-Aware Alignment Extension. :12.

19. Berger SA, Stamatakis A. Aligning short reads to reference alignments and trees. Bioinformatics. 2011;27:2068–75.

20. Berger SA, Krompass D, Stamatakis A. Performance, Accuracy, and Web Server for Evolutionary Placement of Short Sequence Reads under Maximum Likelihood. Syst Biol. 2011;60:291–302.

21. Matsen FA, Hoffman NG, Gallagher A, Stamatakis A. A Format for Phylogenetic Placements. PLOS ONE. 2012;7:e31009.

22. Ondov BD, Bergman NH, Phillippy AM. Interactive metagenomic visualization in a Web browser. BMC Bioinformatics. 2011;12:385.

23. Brancaccio RN, Robitaille A, Dutta S, Cuenin C, Santare D, Skenders G, et al. Generation of a novel next-generation sequencing-based method for the isolation of new human papillomavirus types. Virology. 2018;520:1–10., D571–D578.
